# The BesMan Learning Platform for Automated Robot Skill Learning

**DOI:** 10.3389/frobt.2018.00043

**Published:** 2018-05-31

**Authors:** Lisa Gutzeit, Alexander Fabisch, Marc Otto, Jan Hendrik Metzen, Jonas Hansen, Frank Kirchner, Elsa Andrea Kirchner

**Affiliations:** ^1^Robotics Research Group, University of Bremen, Bremen, Germany; ^2^Robotics Innovation Center, DFKI GmbH, Bremen, Germany; ^3^Bosch Center for Artificial Intelligence, Robert Bosch GmbH, Renningen, Germany

**Keywords:** robotics, imitiation learning, reinforcement learning, manipulation, behavior segmentation

## Abstract

We describe the BesMan learning platform which allows learning robotic manipulation behavior. It is a stand-alone solution which can be combined with different robotic systems and applications. Behavior that is adaptive to task changes and different target platforms can be learned to solve unforeseen challenges and tasks, which can occur during deployment of a robot. The learning platform is composed of components that deal with preprocessing of human demonstrations, segmenting the demonstrated behavior into basic building blocks, imitation, refinement by means of reinforcement learning, and generalization to related tasks. The core components are evaluated in an empirical study with 10 participants with respect to automation level and time requirements. We show that most of the required steps for transferring skills from humans to robots can be automated and all steps can be performed in reasonable time allowing to apply the learning platform on demand.

## 1. Introduction

Autonomous robotic systems can be deployed in unknown or unpredictable, dynamic environments, e.g., in space, search and rescue, and underwater scenarios. These robots require behaviors to manipulate their environment and will face tasks that have not been thought of during design. It is possible to learn solutions to these unforeseen tasks from humans during operation even though the human operators might be far away.

Learning complex behaviors in these cases at once is time consuming or sometimes impossible. An alternative approach is to learn complex behaviors incrementally: small behavioral building blocks are learned separately and are later on combined during consolidation of the complex behavior by concatenation. This strategy has been observed in studies with rodents (Graybiel, 1998)  and children (Adi-Japha et al., 2008) which indicates that it is efficient.

Learning methods for behavioral blocks often combine various approaches to leverage intuitive knowledge from humans. Most of these fall into the categories imitation learning (IL) and reinforcement learning (RL). A standard approach to learn behaviors is to initialize with a demonstrated movement and then refine the skill with RL. See, for example, Argall et al. (2009) for a survey on imitation learning and Kober and Peters (2012); Deisenroth et al. (2013) for a detailed overview of the state of the art in reinforcement learning. Complete descriptions of robot skill learning frameworks are hardly present in the literature. To our best knowledge the only work that gives a complete overview of a learning architecture and is comparable to the work that we present here has been published by Peters et al. (2012). They do not provide a thorough evaluation in terms of automation level and time consumption to learn new skills. Their work includes IL and RL methods to learn so-called motor primitives as well as generalization methods for these motor primitives and even describes methods to learn operational space control. However, in this work as well as in the majority of similar works the relevant behaviors are directly presented by kinesthetic teaching so that the correspondence problem (Nehaniv and Dautenhahn, 2002) that stems from the kinematic and dynamic differences of the demonstrator and the target system is neglected. In addition, only the relevant behavior is presented or it is not discussed how the relevant part that should be transferred is extracted. In contrast to that, we would like to let a human demonstrate the behavior as naturally as possible. With this approach, the system can be situated in a far away place or environment hostile to man and could still learn from a human demonstration although a direct kinesthetic teaching is not possible or would only be possible indirectly in case that a second identical system would be available. To allow the demonstrator to act naturally, we use behavior segmentation methods and solve the correspondence problem as automatically as possible.

In this paper, we focus on the question whether learning from human demonstrations can be used for realistic robotic manipulation tasks and whether the approach can be highly automated and is fast enough to be considered for solving unforeseen tasks during a system’s application in real scenarios. The presented learning platform provides a robot with human demonstrations of behaviors suited for some specific situation. These demonstrations are autonomously decomposed into atomic building blocks which are learned by means of imitation learning. Based on this, RL and transfer learning are used within the learning platform to adapt and generalize the learned behavioral building blocks. While this is not the scope of the presented work, those building blocks can then form the basis for learning more complex behavior in a life-long learning scenario. The learning platform was developed based on learning from human demonstration since for kinematically complex robotic systems it is typically infeasible to generate behavior from scratch within a robot due to high-dimensional state and action spaces and the limited number of trials a robot can conduct. The presented learning platform will be described and evaluated with respect to its components’ and its overall performance. For this purpose, we will solve the problem of target-directed ball-throwing with a robotic arm.

## 2. Learning Platform

**Figure 1:**
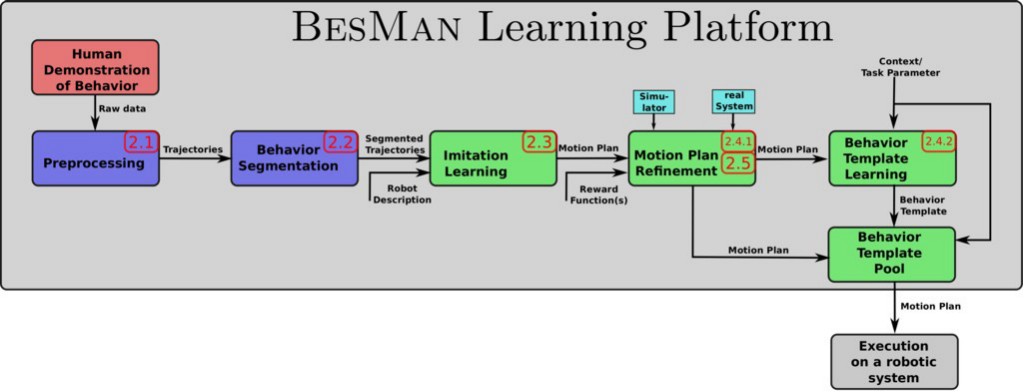
Dataflow diagram of the BesMan Learning Platform. A behavior is demonstrated and segmented into smaller behavioral building blocks. Motion plans corresponding to these building blocks are imitated and refined using reinforcement learning and/or transfer learning. Acquired motion plans are specific for a task but can be generalized to more generic behavior templates. Once a new specific task is encountered, the behavior template is instantiated and yields a task-specific motion plan. Numbers show the corresponding sections in this article that explain the module in detail.

This section describes the architecture of the learning platform (see Figure 1) which was developed within the project BesMan^1^ to transfer movement behavior from a human demonstrator to a robotic system. During this process so-called motion plans and behavior templates are generated. Motion plans represent solutions to generate specific behaviors and behavior templates represent generic movements to generate a flexible behavior able to, e.g., reach different points in space and to be executed on different systems with different morphology.

Demonstrations are recorded and preprocessed as described in Section 2.1. The “Behavior Segmentation” will decompose demonstrations into simple behavioral building blocks. Segments that belong to the same type of movement are grouped together to obtain multiple demonstrations for the same motion plan. Details of the behavior segmentation and classification approach are described in Section 2.2. This part of the learning platform generates labeled demonstrated behavioral building blocks that are independent of the target system.

For each relevant segment, IL methods are used to represent the recorded trajectory segments as motion plans (see Section 2.3). Motion plans describe trajectories that could be executed by the robot and mimic the trajectories presented by the human demonstrator during a single behavioral building block. However, human demonstrators usually have considerably different kinematic and dynamic properties in comparison to the robotic target systems, hence, these motion plans might not produce the same result on the target system. To account for this, the “Motion Plan Refinement” module can use RL (see Section 2.4) to adapt the motion plan. This requires interaction with the real or simulated target system and the specification of a reward function which tells the learning algorithm how well a motion plan solves the task. Alternatively or in conjunction with RL, transfer learning as described in Section 2.5 can be used to adapt motion plans. Using this method, differences between learned behaviors in simulation and on the real robot are also considered during the motion plan refinement.

Motion plans are solutions for very specific settings. It is often necessary to learn more generic behavior templates that can be applied to similar settings. This is achieved by the “Behavior Template Learning” module (see Section 2.4). Behavior templates are capable of generating motion plans for new but similar settings. Once a behavior template has been learned, it is added to the “Behavior Template Pool” which is accessible from the robotic system. The behavior templates in this pool can be used directly during online operation.

In the following sections, detailed descriptions of the modules are given.

### 2.1. Behavior Acquisition and Preprocessing

The movements of the demonstrator that should be transferred to a robotic system are recorded using a Qualisys motion capture system. Visual markers are attached to the human demonstrator and to objects which are involved in a particular task. In this way, movements and important changes in the environment can be recorded at a high accuracy.

**Figure 2:**
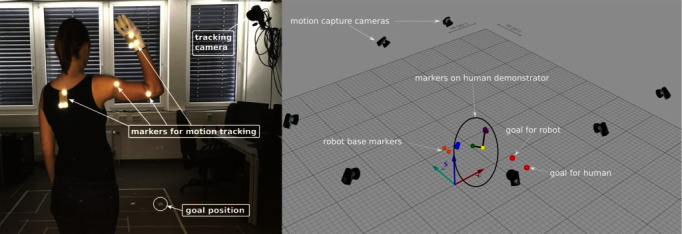
Data acquisition setup. Motion capture cameras, markers, and goal positions for the throws in the setting are displayed. Written and informed consent for publication of the photo has been obtained from the depicted individual.

Markers are placed at shoulder, elbow, and hand of the human demonstrator. Three markers at one position can be used to infer orientations. This is important in manipulation task which require the robot to imitate the orientation of the hand. By placing three markers at the back, all marker positions can be transformed into a coordinate system relative to the demonstrator to make the recordings independent from the global coordinate system of the camera setup. Additional markers can be placed on manipulated objects. An example of a recording setup for ball-throwing behaviors is shown in Figure 2.

Because a passive marker-based motion-capture system is used, we implemented an automatic marker identification based on the relative positions of the markers to each other. Similar methods are provided by manufacturers of motion capture systems, for example, the Qualisys track manager^2^ offers “automatic identification of markers” which is based on previously labeled motion capture data. Other works rely on manually defined or otherwise inferred skeletons. Refer to Meyer et al. (2014); Schubert et al. (2015, 2016), for details about some of these approaches.

### 2.2. Behavior Segmentation

In the recorded demonstrations the main movement segments have to be identified to transfer the movements to a robotic system. We segment the behavior into its main building blocks and classify each building block into a known movement class. By using an automatic approach, movement sequences needed to solve a certain task with a robotic system can easily be selected without manual user interference.

To decompose demonstrated behaviors into simple building blocks, we have to identify the characteristics of the movements. Manipulation movements usually have bell-shaped velocity profiles (Morasso, 1981). Based on this knowledge, we developed the velocity-based Multiple Change-point Inference (vMCI) algorithm for the segmentation of manipulation behavior (Senger et al., 2014). The vMCI algorithm is an extension of the Multiple Change-point Inference presented by Fearnhead and Liu (2007), in which Bayesian inference is used to determine segment borders in a time series. We extended this algorithm to detect building blocks in human manipulation movement (Senger et al., 2014). Each segment is represented with a linear regression model. In the vMCI algorithm, the velocity of the data is independently modeled with a bell-shaped basis function. The algorithm identifies segment borders at positions where the underlying model of the data changes. These borders can be determined using an online Viterbi-algorithm in a fully unsupervised manner. By including the velocity into the inference in the vMCI algorithm, segments with a bell-shaped velocity can be detected automatically without need for parameter tuning, as shown in several experiments (Senger et al., 2014; Gutzeit and Kirchner, 2016).

Because in general there is no ground truth segmentation available for human movements, the vMCI algorithm has been compared to other segmentation algorithms on synthetic data generated from sequenced dynamical movement primitives [DMP; (Ijspeert et al., 2013)] as well as on real human manipulation movements (Senger et al., 2014). It has been shown, that the vMCI algorithm is very robust against noise and to the selected parameters. These are very important properties because noise in the data as well as differences in the movement execution can be handled. Furthermore, the manual user input needed can be kept low.

Resulting segments need to be annotated in order to select movement sequences that should be learned and transferred to the robot. To minimize manual effort, this annotation should work with small training data sizes. As detailed in Gutzeit and Kirchner (2016), we use a k-Nearest Neighbor (k-NN) classifier with Euclidean distance as distance metric on the normalized trajectories transformed to a coordinate system relative to the human demonstrator to assign predefined movement classes to the acquired segments. The k-NN algorithm is chosen because it has only one parameter, k, which needs to be selected. No further parameter tuning is needed. We choose k=1, because we want to classify the segments with a low number of training examples. A higher k could result in a higher number of miss-classifications using very small training set sizes. We showed, that using this simple Euclidean based classification algorithm, it is possible to classify human movements into different movement classes at a high accuracy.

### 2.3. Imitation Learning

We use imitation learning [IL; Schaal (1997)] to obtain motion plans from the recorded trajectories. A workflow for learning from demonstrations must address the correspondence problem as well as the representation of the motion plan. In this section we introduce motion plan representations used within the BESMAN learning platform and discuss the correspondence problem.

#### 2.3.1. Motion Plan Representation

One of the currently most popular motion plan representations to imitate movements are so-called dynamical movement primitives (Ijspeert et al., 2013). The DMP representation has a unique closed-form solution for IL which makes it very appealing for our purpose. After imitation, the parameters of the DMP can be adjusted easily which makes it suitable for policy search (see Section 2.4.1). The standard DMP formulation allows to set the initial state, goal state, and execution time as meta-parameters. The DMP converges to a velocity of 0 at the end of a discrete movement.

There are several variants of DMPs. An extension that allows to set a goal velocity has been developed by Mülling et al. (2011, 2013). We use this DMP formulation for trajectories in joint space. Representing three-dimensional orientations in a DMP is not straightforward. A solution has been proposed by Pastor et al. (2009) and improved by Ude et al. (2014) through correct handling of orientation represented by unit quaternions. To represent trajectories in Cartesian space, we use a combination of a position DMP as formulated by Mülling et al. (2011, 2013) and an orientation DMP by Ude et al. (2014).

#### 2.3.2. Correspondence Problem

Demonstrated actions must be executable by the target system to apply IL. Is is not possible to directly transfer joint angles from a human arm to a robot arm because it has different joints, degrees of freedom, and link lengths. The reason is the correspondence problem (Nehaniv and Dautenhahn, 2002), which consists of two subproblems (Argall et al., 2009). The following mappings have to be defined: the record mapping, which maps marker trajectories to a sequence of actions or system states, and the embodiment mapping, which maps the recorded sequence to a trajectory that is executable on the target system.

The record mapping $g_{R}:A_{T}\to D$ maps from some not directly observable space $A_{T}$ in which the teacher performs the demonstration (e.g., joint angles of a human, muscle activity, applied torque, etc.) to a corresponding observation space D. We cannot directly observe the actions of the agent but we can observe the marker positions, which means that a part of the record mapping is already given. Instead of using the observed marker positions directly to represent D, we reduce the marker positions to a representation that is more meaningful to describe manipulation behavior and is independent of the platform: we use observed marker positions to extract end-effector poses in some reference frame, that is D⊂SE(3). The calculation of end-effector poses from observed marker positions is specific for a marker setup and has to be defined. The reference frame depends on the application. For goal-directed manipulation behavior Wirkus (2014) proposes to use the target as a reference frame, e.g., a box that we want to grasp. For behaviors like ball-throwing it is better to use a reference frame on the teacher, e.g., the back, because the target object (the ball) will be moved with the end-effector. Works by Niekum et al. (2015); Calinon (2016); Manschitz et al. (2016) select the reference frame automatically but we did not consider this here.

The embodiment mapping $g_{E}:D\to A_{L}$ maps from the observations d∈D to a corresponding action $a\in A_{L}$ that the learner has to perform to achieve a similar result. It is specific for the task and the target system. Although one might think that transfering end-effector poses to the target system is simply an inverse kinematics problem, it can actually be much more complicated: the target system might not have the same workspace, kinematic structure, and dynamic capabilities as the teacher. Hence, we propose to use multiple methods sequentially to determine the embodiment mapping automatically:

- Black-box optimization to determine synchronization frames,
- Spatial and temporal scaling to take the kinematic and dynamic capabilities of the target system into account,
- And refinement with policy search.

The first two methods will only guarantee that the actions are executable on the target system. That does not mean that the result of the actions actually produces the same effects that have been observed in the demonstration by the human and the task is solved. Not all of these methods are required for all categories of tasks. We will now explain and discuss when to use these methods.

**Figure 3:**
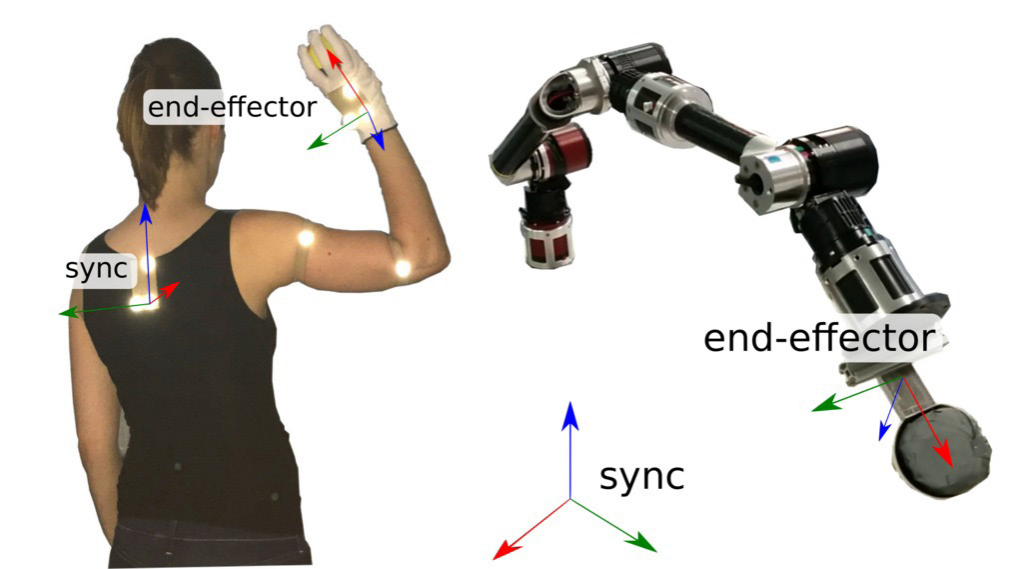
Synchronization frames on the demonstrator and on the target system. Written and informed consent for publication of the photo has been obtained from the depicted individual.

The reference frame of our target system is a so-called synchronization frame, that is, we use it as the base frame in which the demonstration is performed [see Bongardt (2015) for a detailed introduction of this term]. It is denoted as ${\left[P_{T\left(b\right)}\right]}^{S}\in SE\left(3\right),$ where T(b) denotes the base frame of the target system. For simplicity, we assume that the synchronization frame is constant over time. In some situations it is obvious how the corresponding reference frame in the target system should be selected to transfer demonstrated motion plans in Cartesian space. For example, if we define the teachers reference frame to be the target of a grasping movement, we will not change this in the target system. In other cases it is not so obvious. Consider a ball-throwing movement where the reference system of the recorded movement is the human teacher’s back (see Figure 3). When we want to transfer the observed motion plan to a robotic arm, it is not obvious where we would put the synchronization frame. We can choose it arbitrarily.

We can use this freedom to account already for some of the problems that occur in the embodiment mapping. Without having any information about the structure of the task, we can optimize the synchronization frame so that we can find a joint configuration for each end-effector pose from the demonstration so that velocity, acceleration, and/or jerk of the trajectory is minimized. The first objective ensures that the trajectory is mapped into the robot’s workspace and the other objectives account for kinematic and dynamic problems that might occur. Otherwise it happens quite often that inverse kinematic solvers run into local optima during the execution of the motion so that big changes in the joint configuration occur during the execution. Even if that is not the case, it might happen that a small displacement in Cartesian space that requires almost no effort by the teacher might result in a large displacement in the joint space of the target system.

The only information that we have to give at this point is a kinematic description of the target system. To find a locally optimal solution to our problem, we can use any black-box optimization algorithm like CMA-ES (Hansen and Ostermeier, 2001) or L-BFGS (Nocedal, 1980).

For a specific task on the target system we have to scale the demonstrated trajectory spatially by setting the start and goal state of the trajectory. From the inverse kinematics we can then compute the required velocities in joint space. To keep the velocities within the limits of what is achievable by the target system, we can do a temporal scaling of the movement, e.g., by interpolation. Temporal scaling is simple when we represent the demonstrated trajectory as a DMP because the execution time is a meta-parameter of the DMP.

Until now we only integrated knowledge about our target system into the embodiment mapping. To ensure that the imitated skill has the same effects as the demonstration, we must integrate knowledge about the task. This will be done in the policy refinement step. We have to define a reward function that can be used by reinforcement learning methods to complete the embodiment mapping. Here we account for kinematic and dynamic differences that cannot be resolved easily, e.g., a human teacher might have a hand structure that is different from the target system. An example is displayed in Figure 3: the target system does not even have an active hand. It has a scoop mounted on the tip of the arm. In other cases, the robot might have a gripper that does not have all of the capabilities of a human hand, e.g., a parallel gripper. Another problem in the ball-throwing domain is the dynamic and kinematic difference between the human demonstrator and the target system. It might be possible for the robot to execute the throwing movement after temporal scaling but this step can reduce the velocity in Cartesian space so drastically that the ball does not even leave the scoop any more. The methods that can be used to solve these problems are discussed in Sections 2.4.1 and 2.5.

### 2.4. Reinforcement Learning

The learning platform provides tools to adjust motion plans to specific target systems and to generalize motion plans over specified task parameters based on reinforcement learning [RL; Sutton  and Barto  (1998)].

Depending on the application, we have to decide whether learning will take place in simulation or in reality. Learning in reality would give the best results, however, this might not be feasible because it takes a lot more time and usually requires human assistance. Furthermore, the robot could potentially damage itself. The more complex a robot is the more parts can break and, hence, the more fragile it becomes. It is often a good idea to model the relevant aspects of the task in simulation and start with the refinement in simulation. When a good motion plan is obtained, this can be used to start learning in reality directly or approaches can be applied to handle the simulation-reality gap (see Section 2.5).

We decided to focus on policy search and its extensions. A good overview is provided by Deisenroth et al., 2013. Policy search is very sample-efficient in domains where a good initial policy can be provided, the state space is high-dimensional and continuous, and the optimal policy can be represented easily with a pre-structured policy such as a DMP.

It depends on the target system and the application which policy searchs methods should be used. Standard policy search (see Section 2.4.1) is always included in our learning platform to ensure that the embodiment mapping is completed. We can generalize the obtained motion plan to a behavior template that takes the current context as a parameter to modify its motion plan using contextual policy search (see Section 2.4.2). In an active learning setting the generalization can be more effective (see Section 2.4.3).

A more recent alternative to these policy search methods that is gaining more and more popularity in the reinforcement learning community is end-to-end learning with deep reinforcement learning (Arulkumaran et al., 2017). Deep RL uses complex policies or value functions, represented by neural networks. The benefit is that it allows a conceptually easy integration of sensors like cameras. Using a neural network, however, complicates the integration of prior knowledge from non-experts through imitation learning. Movement primitives and policy search are very appealing methods from a robotics perspective, since just one demonstration is enough to learn motion plan that can be refined by policy search.

#### 2.4.1. Policy Search

In policy search we modify a parameter vector θ, which can include the parameters and/or meta-parameters of the motion plan π, so that the reward $R_{\theta }$ is maximized. Depending on wether we want to optimize only a few meta-parameters or the whole set of parameters, we can select the policy search method. We use several algorithms in the learning platform. Covariance matrix adaption evolution strategy [CMA-ES; Hansen and Ostermeier (2001)] is a black-box optimization algorithm which can be used to optimize any parameterized policy (Heidrich-Meisner and Igel, 2008). Unlike CMA-ES, Relative Entropy Policy Search [REPS; Peters et al. (2010)] limits the information loss during exploration. Path Integral Policy Improvement [PI${}^{2}$; Theodorou et al. (2010)] is based on stochastic optimal control. It requires to specify an initial policy and a covariance matrix which governs exploration in weight space. All of these methods are local search approaches which means they need a good initialization provided through imitation learning. Bayesian Optimization (Brochu et al., 2010) is a global optimization method. It has been shown to be applicable to policy search (Calandra et al., 2016). Bayesian optimization is usually limited to a few parameters because of the computational complexity, however, it is very sample-efficient.

#### 2.4.2. Contextual Policy Search

A disadvantage of using prestructured motion plans like DMPs is that they are designed for a specific situation and can only generalize over predefined meta-parameters. They are not able to generalize over arbitrary task parameters that often have non-trivial relations to the optimal motion plan. This is addressed by contextual policy search (Deisenroth et al., 2013) which is an extension of policy search. Contextual policy search learns a so-called upper-level policy θ=π(s), which is a mapping from a context vector s that describes the task to the optimum parameter vector θ of an underlying motion plan.

We implemented Contextual Relative Entropy Policy Search [C-REPS; Kupcsik et al. (2013)], developed an extension called active training set selection C-REPS [aC-REPS; Fabisch et al. (2015)], and we developed Bayesian Optimization for Contextual Policy Search [BO-CPS; Metzen (2015)]. Our method BO-CPS Metzen (2015) is based on Bayesian optimization and is much more sample-efficient than the local search approach C-REPS but it does not scale well to a large number of parameters.

#### 2.4.3. Active Learning

Active contextual policy search extends contextual policy search for cases in which the learning agent is able to determine the context it wants to explore. This is the case for example in the ball-throwing domain. In this setting it is desirable to select the context s that maximizes the learning progress in each episode. We have shown that selecting the context gives an advantage in combination with C-REPS (Fabisch and Metzen, 2014) although we only used a discrete set of contexts and modeled context selection as a non-stationary multi-armed bandit problem. We developed an active context-selection approach for BO-CPS based on entropy search (Hennig and Schuler, 2012), which is called Active Contextual Entropy Search [ACES; Metzen (2015)]. ACES allow to select from a continuous set of contexts. We also developed minimum regret search [MRS; Metzen (2016)], which a novel exploration strategy for Bayesian optimization (more specifically: an acquisition function). In contrast to entropy search, which aims at maximizing the information gain about the optimum, MRS aims at minimizing the expected regret of its final recommendation. MRS explores more globally and is less likely to focus prematurely on a local optimum.

### 2.5. Simulation-Reality-Transfer

Motion plans learned only in simulation often perform worse when they are executed in reality. In certain situations, this performance drop is small, for example, in open-loop control with a robot having accurate and precise actuators. In such a case, it can be sufficient to apply policy search methods in simulation and transfer the result directly to the real system. Using closed-loop control while having sensor and actuator noise, deformation, fatigue and other factors contributing to the disparity between simulation and reality, the *Reality Gap* becomes a problem.

A wide range of approaches to this issue have been proposed, many of which aim to improve the physical correctness of the simulation (Jakobi et al., 1995,  1997; Bongard and Lipson, 2005) or adaptation capabilities (Urzelai and Floreano, 2001; Hartland and Bredeche, 2006). More recently (Koos et al., 2013) and (Cully et al., 2015) have applied behavioral descriptions of the controller to be optimized to build and update an internal model guiding the search towards controllers that work well in reality without adapting the original simulation model.

To choose an appropriate algorithm, one has to consider several factors, including: the task complexity, the consistency of the robot and the environment, the complexity of the environmental interactions and required precision of the model as well as the availability of the robot. The cost and robustness of the robot determines how often and what type of tests (potentially risky movements) can be performed in reality.

For example, the ball-throwing task in the current work requires to release the ball at a certain position and velocity. Both depend on the end-effector trajectory and the time of release. The time of release, speed and direction are not easy to predict (Otto, 2015). The development of an accurate model of the physical interactions of the ball and the ball mount would require a detailed analysis of the ball as well as the ball mount. We want to reduce the amount of expert knowledge or additional testing and solve the task with minimum user input. Hence, we choose to apply the Transferability Approach by (Koos et al., 2013), which minimizes the number of tests on the real system by focusing on the task to be solved rather than optimizing simulation accuracy.

The Transferability Approachis based on the hypotheses that the performance of a specific behavior on the target system mainly depends on (1) its performance in simulation and (2) the correctness of the simulation for this behavior. Thus, the optimization problem can be reformulated to find actions maximizing the reward in simulation and the *transferability*. While the simulation model remains unaltered, the motion plans are adjusted to approximate the Pareto front (optimal trade-off solutions) for both criteria.

Motion plans generated by imitation learning constitute the initial population for the optimization. Motion plans are evaluated in the simulation while features are extracted to compute the objective functions. By iterative variation and selection through evolutionary operators, the motion plans are optimized. In regular intervals, an update heuristic is used to select motion plans to be transferred based on their *behavioral diversity*. The behavioral diversity is a quantity that describes how much an observable activity differs from a set of other activities. The data recorded in these transfer experiments is compared with the corresponding data in simulation and their disparity is stored for each transferred motion plan as the simulation-to-reality disparity. Most motion plans are not transferred. Instead, a *surrogate model* is used to estimate the disparity by interpolating between the observations.

## 3. Evaluation of the Learning Platform

Most of the individual modules of the learning platform have already been evaluated in previous works (Fabisch and Metzen, 2014; Senger et al., 2014; Fabisch et al., 2015; Metzen, 2015; Metzen et al., 2015). In this section, we evaluate the learning platform as a whole in a ball-throwing scenario. We transfer the human movements to a robotic arm. Furthermore, we evaluate the learning platform with respect to time requirements and level of automation. We show that the learning platform can be run with minimal user interference by different non-expert subjects which demonstrated the ball-throw.

### 3.1. Methods

In the following we describe the applied methods for learning a ball-throwing behavior from humans and for transferring it to a robotic arm.

#### 3.1.1. Robotic System

We transfer the movements to the robotic arm COMPI (Bargsten and de Gea Fernandez, 2015) displayed in Figure 3. A scoop that can hold a ball is mounted as COMPI’s end-effector. The position where the ball hits the ground can not always be identical because of varying positions of the ball in the scoop, varying shape of the deformable and not perfectly round ball, inaccuracies in the execution of the desired trajectory, and measurement errors. How reproducible this position is depends on the throwing movement. For some throwing movements the SD of the position can be more than a meter because the ball sometimes falls down before the throwing movement is finished and sometimes not. To estimate the maximum reachable accuracy, we designed 4 throws manually for which we measured the SD of the touchdown position in 20 experiments per throw. The mean positions were 1.3 to 2.3 m away from COMPI. The standard deviations were about 4.5 to 7 cm. To measure the position where the ball hits the ground we use the motion capture system and a ball that is recognized as a marker.

#### 3.1.2. Data Acquisition

The setup used to record the demonstration of a throw can be seen in Figure 2. Seven cameras tracked eight visual markers attached on the human and the target area. Only five cameras were focused directly on the subject. The recorded marker positions were labeled according to their position on the human body (e.g., “shoulder”). The subjects had to throw a ball to a goal position on the ground, approximately 2 m away. To limit the range of possible throws, they were instructed to throw the ball from above, i.e., the hand is above the shoulder while throwing (see Figure 2). The subjects had to move their arm to a resting position in which it loosely hangs down between the throws. The movement was demonstrated by 10 subjects. All subjects were right-handed and had different throwing skills ranging from non-experts to subjects performing ball sports in their free time, like basketball, volleyball, or handball. Each subject demonstrated 8 throws in 3 experiments which results in total numbers of 24 throws per subject, 30 experiments, and 240 throws for all subjects.

#### 3.1.3. Segmentation and Movement Classification

Based on the position and velocity of the hand, the recorded demonstrations were segmented using vMCI. The determined segments can be assigned into 4 different movement classes: strike_out, throw, swing_out and idle. To classify the resulting segments into predefined movement categories, we use a 1-NN classifier as described in section 2.2.

#### 3.1.4. Imitation Learning

IL is based on end-effector poses. End-effector trajectories cannot be transferred directly to the robot’s coordinate system so that we have to do a synchronization frame optimization to translate and rotate the original trajectory so that it fits into the workspace of COMPI. The end-effector trajectories are transformed into joint trajectories via inverse kinematics. In addition, we scale the joint trajectories so that the joint velocity limits of the target system are respected. In a last step, the throwing movement is represented as a joint space DMP via IL. Moreover, a minimum execution time of 0.95 s is set to reduce the velocity and accelerations, which are penalized during the following optimization.

#### 3.1.5. Motion Plan Refinement

While the DMPs resulting from IL can be executed on the robot, they do not necessarily have the same effect on the ball as the movements of the human. The lack of actuated fingers as well as kinematic and dynamic differences to the human lead to the need for adaptation, which we do via policy search. The adapted policy parameters include: initial position, goal, weights and execution time, consisting of 6, 6, 36 and 1 value(s), respectively. Following the concept of the Transferability Approach, we aim to minimize two objectives: (a) the target distance of the touchdown position in simulation and (b) the distance between the touchdown position in simulation and in reality. The target distance in reality is not directly optimized, but evaluated during and at the end of the experiment.

**Figure 4:**
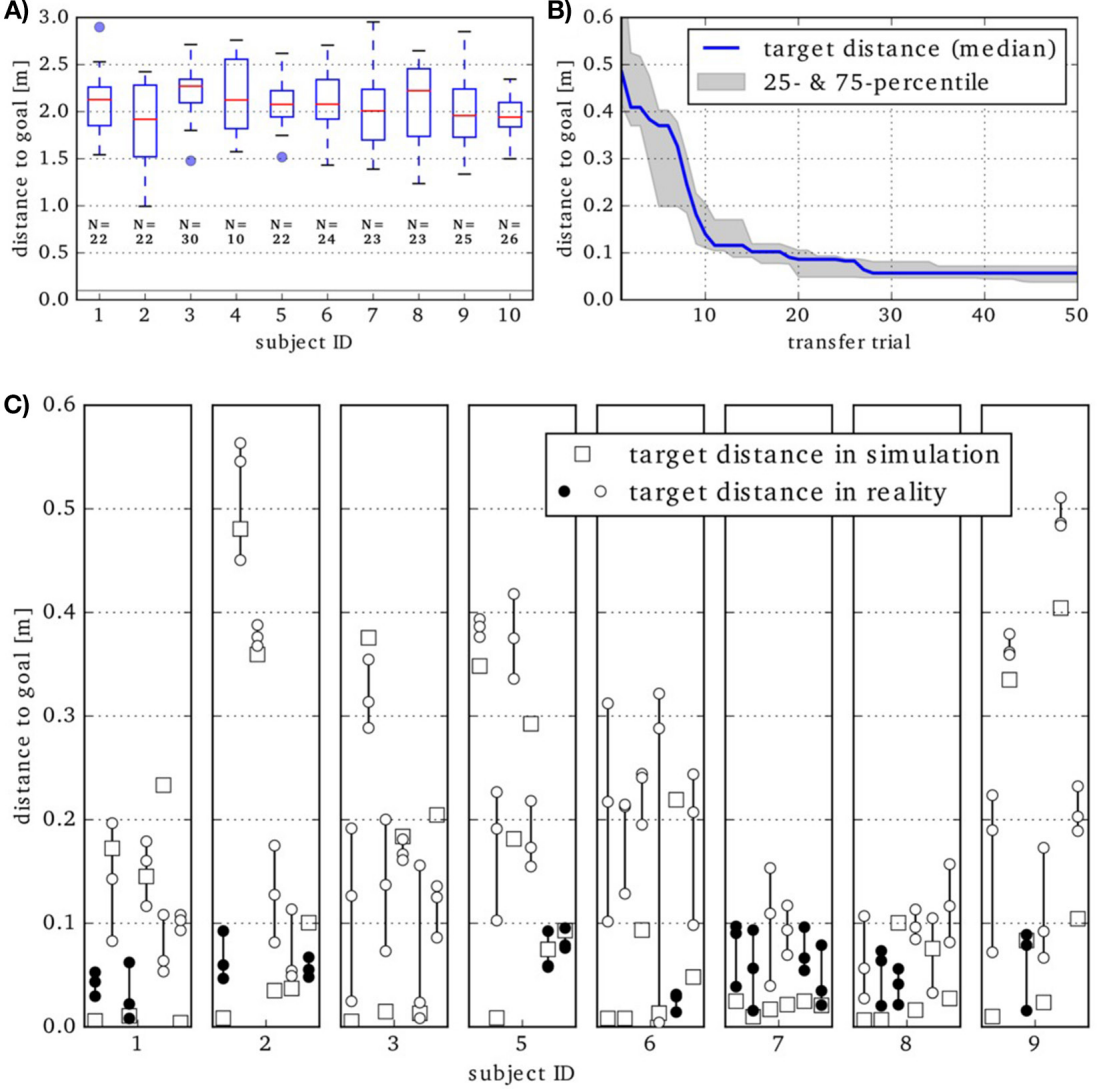
**(A)** Results of IL evaluated in simulation are shown by Tukey boxplots. N is the number of throw segments detected in the data of a specific subject. The solid gray line indicates the 0.1 m threshold. Please note the different scaling of the ordinate, compared to **(B)** and **(C)**. **(B)** Closest distance to target achieved in transfer trials during robot-in-the-loop optimization. The median over 8 subjects of the best results so far is shown. For each subject, 50 transfer trials are executed on the robot during the optimization. **(C)** At the end of the optimization process, for each subject, six motion plans are automatically selected for evaluation. The result from the deterministic simulation is shown by squares. Each motion plan is evaluted three times in reality (circles). If the ball landed closer than 0.1 m to the target in all three repetitions, the circles are filled, otherwise not.

The optimization consists of several steps. (1) **Refinement in Simulation**: Results from IL are optimized to throw at least 0.1 m (see gray line in Figure 4A) close to the target [objective (a)]. Per subject we perform 6 optimization runs using all available motion plans in each run. After a maximum of 10,000 episodes (500 generations with a population size of 20), the optimization is stopped. (2) **Refinement in Simulation and Reality**: The Transferability Approachis performed until 25 transfer experiments are executed on the robot. The initial population consists of successful throwing movements from the optimization in simulation (previous step). They are optimized to minimize both target distance, position and velocity limit violation as well as an acceleration penalty. After 50 episodes (corresponds to population size) in simulation, we do one transfer. (3) **Simulation refinement**: The simulation is optimized to replicate the actual results of the 25 transfer experiments [objective (b)]. Two parameters, the ball-release angle that influences the time of the ball release from the robot end-effector and the height of the robot are adjusted via a simple grid search. (4) **Refinement in Simulation and Reality**: The Transferability Approachis performed with the same initial conditions as in step 2 until 50 (additional) transfer experiments are executed on the robot (objectives a and b) (5) **Evaluation of Candidates**: Several candidate solutions are executed in reality with 3 repetitions to determine a final performance with regard to the target distance and repeatability.

The Transferability Approachis performed until 25 transfer experiments are executed on the robot. The initial population consists of successful throwing movements from the optimization in simulation (previous step).

To infer the transferability of a motion plan that is not tested in reality, the surrogate model requires a low dimensional description of the *type of activity* that is created by the motion plan in simulation. We use a subset of the simulation results to describe the activity by 17 features including (among others) the time of release, maximum joint accelerations and the posture at half-time. After each transfer, the observed touchdown-disparity and the activity description computed in simulation are appended to the observations stored in the surrogate model.

#### 3.1.6. Required Time and Level of Automation

For each main module of the learning platform that is needed to learn a new behavior, the required time was measured. The time required to learn a new behavior is strongly influenced by the degree of automation, which is furthermore relevant to the easiness of application. Therefore we evaluated the degree of automation for each module of the learning platform.

### 3.2. Results and Discussion

We evaluate two parts of the learning platform separately. The first part is independent of the target system and includes data acquisition, preprocessing, and segmentation (see Section 3.2.1). The second part depends on the target system and includes imitation and refinement (see Section 3.2.2). The whole learning platform will be evaluated with respect to required time (see Section 3.2.3) and level of automation (see Section 3.2.4).

#### 3.2.1. Segmentation and Movement Classification

The automatic marker labeling could not always be used for the recordings. The reason for that are gaps in the recordings. Because we had to cover a large area (target area and subject’s workspace) it was nearly impossible to record every marker all the time for every subject with only seven cameras. With a smaller volume that has to be covered, more cameras, or active markers this problem could be solved.

The vMCI algorithm successfully detected movement segments with a bell-shaped velocity profile in the demonstrations. Exemplary segmentation results of this data can be found in (Gutzeit and Kirchner, 2016). No manual intervention was needed for this segmentation step because the vMCI determines the movement segment borders unsupervised and its parameters can be computed directly from the data (Senger et al., 2014).

To classify the determined segments with 1-NN, a training set of 40 throwing demonstration from subjects 1–5 (one experiment from each subject) is manually labeled. The throwing movements of these 5 subjects that have not been used for training (two experiments per subject) and all the throwing movements of subjects 6–10 (three experiments per subject) are used to evaluate the classifier. On the test set consisting of the remaining demonstrations of subjects 1–5, an accuracy of 93.2% could be achieved. The important movement class throw, which should be transferred to the robotic system, was detected with an accuracy of 98.6% on this test set. The second test set consisted of demonstrations of subjects 6–10, i.e., no throwing movements of these subjects were used to train the classifier. On this data, the accuracy was 89.1 and the class throw was detected with 93.9% accuracy. As a conclusion, different movement classes can be successfully recognized based on a simple Euclidean based 1-NN using only marker positions on arm and hand as features. Even movements with considerable differences in execution, like in the ball-throwing scenario, the 1-NN based classification is successful.

#### 3.2.2. Imitation Learning and Motion Plan Refinement

In this section, we evaluate the target distance measured at several steps of the learning platform.

(0) Initial performance: At first, we evaluate the results of the IL in simulation. The IL does not consider suitability for holding and throwing the ball. Hence, several motion plans result in a simple ball drop near the initial position. This is reflected by target distances around 2.15 m in (Figure 4A). None of the simulated ball throws is closer than 1 m to the target. One may also note the variation in the number of throw segments that are detected in the data containing 24 actual demonstrations per subject. (1) Refinement in Simulation: For one subject, the goal (distance is below the 0.1 m threshold indicated by the gray line in Figure 4A) was not reached. For all remaining subjects, the goal was reached in less than 2,200 episodes in at least one of the 6 runs. (2) **Refinement in Simulation and Reality**: For 2 out of 9 subjects, the transfer experiments reached target distances below 0.1 m in reality. The deviations of the touchdown positions in reality and in simulation seem to be systematic, i.e., for throwing behaviors resulting from the same subject, similar deviations are found. Having a constant offset between simulated and real results contradicts the premise of the Transferability Approach, aiming to find a region in the parameter space that features transferable motion plans. Hence, we decide to adapt the simulation specifically to predict the touchdown positions for some of the movements more accurately. (3) **Simulation refinement**: To reduce the offset of real and simulated results, we minimize the median of the touchdown-disparities obtained for the 25 transfer experiments so far. This is done via a simple grid search on the value for the robot height and the ball-release angle. The medians could be reduced to a range of 0.11 to 0.23 m (depending on subject; compared to 0.23 to 0.73 m before). (4) Refinement in Simulation and Reality: For one subject, this optimization step was aborted after 10 critical transfer experiments, during which joint limits were exceeded and the robot was deactivated consequently. For all of the remaining subjects, target distances below 0.1 m as well as touchdown-disparities below 0.1 m occurred in the 50 transfer experiments. The best target distances so far are shown in Figure 4B). The curve indicates that 25 transfers are sufficient to minimize the target distance. (5) **Evaluation of Candidates**: Figure 4C) shows the performance evaluation of 6 automatically selected final candidate solutions (2 with the lowest target distance in reality and 4 from the Pareto front. Up to 4 of these hit the ground *reliably*, *i.e.*, the target distance is below 0.1 m in all three repetitions (marked by filled dots). For seven out of eight (remaining) subjects, at least one selected candidate solution hits the target reliably.

#### 3.2.3. Required Time

An overview of the required time for each step can be found in Table 1. Note that labeling the dataset for movement classification usually has to be done only once. The required time for successful automatic marker labeling is much faster than manual labeling even though the automatic labeling is slow because of the bad quality of the data (see 3.2.1). If the markers were always visible the automatic labeling would have taken only about some seconds. The longest part in the whole process is the refinement for the target platform (imitation, policy search, transfer), which is a difficult problem that involves interaction with the real world.

**Table 1:** Required time (per experiment, 8 throws) for each stage of the learning platform.

Step	Time	Automated	Required knowledge
Attaching markers for motion capture	0:55 min	**×**	
Motion capture	1:08 min	**×**	
Automatic marker labeling	4:58 min	✓	Neighboring markers, initial pose
Manual marker labeling	9:19 min	**×**	
Behavior segmentation	0:44 min	✓	
Labeling for movement classification^*^	50 min	**×**	
Movement classification	2 sec	✓	
Imitation learning	4:20 min	✓	Robot description
Policy search	10 min	✓	Reward function, simulation
Transferability approach	75 min	(✓)	Reward function, simulation

* Dataset from 5 experiments including 40 throws

#### 3.2.4. Level of Automation

Although we have automated the process of acquiring new behaviors, still some human intervention is required either through knowledge that has to be given to the system or by interacting physically with the system. An overview can be found in Table 1.

Of course it is necessary that a human demonstrates the movement. The labeling of the markers can be completely automated with more cameras. However, we could not achieve the maximum possible level of automation in our experiments. Movement classification requires a dataset that is labeled manually but we minimized the effort by using a classifier which classifies at a high accuracy with small training data sets. When we set up the learning platform for a new target system and type of manipulation behavior we have to decide which components we combine for embodiment mapping, e.g., in the ball-throwing scenario the synchronization frame optimization was useful which is not always the case. However, the IL is completely automated. The motion plan refinement with the transferability approach requires human assistance because the robot has to try throwing movements in the real world and is not able to get the ball back on its own. The process itself is automated so that no knowledge about the system or the task is required from the human at this step. In addition, we have to define a reward function that describes how a solution of a task should look like and because we want to minimize the interaction with the real world we use a simulation which has to be designed. This is a manual process at the moment.

## 4. Evaluation of Behavior Template Learning

In separate experiments, we evaluated the component “Behavior Template Learning” in the ball-throwing scenario. It is irrelevant how the initial throw has been generated and, hence, it is not required to evaluate the component for each subject. The behavior template is learned directly in reality without any simulation. Similar but not directly comparable work for ball-throwing has been published by da Silva et al. (2014) and Ude et al. (2010) and for table tennis by Mülling et al. (2013).

### 4.1. Methods

#### 4.1.1. Experimental Setup

From an initial motion plan, we generalize to a behavior template that can hit any target s (context) in the area [1m,2.5m]×[−1m,1m], where COMPI is located at (0m,0m). The performance is evaluated on a grid of 16 test contexts {−0.7,−0.2,0.2,0.7}×{1.3,1.6,1.9,2.2} that will not be explored during the training.

#### 4.1.2. Behavior Template Learning

In order to apply the most sample-efficient algorithm (BO-CPS) we had to reduce the number of parameters of the motion plan drastically. Otherwise the computational complexity of the problem would be too high. It is not possible to learn all weights of a typical joint space DMP, e.g., ten weights per joint would result in 60 parameters. For that reason we selected only two meta-parameters that were optimized: the execution time and the goal position of the first joint. The execution time will let us vary how far the ball is thrown and the goal position of the first joint will determine the angle of the throw. Note that there is not a direct linear relation between the goal position of the first joint and the throwing angle because of a complex interaction between the deformable ball and the scoop. It depends on the execution time as well. The goal is to learn an upper level policy that predicts a close to optimal pair of execution time and goal position of the first link for a given target so that a specific motion plan can be generated to hit this target. We conduct a thorough evaluation that compares the acquisition functions upper confidence bound and entropy search for BO-CPS.

### 4.2. Results and Discussion

**Figure 5:**
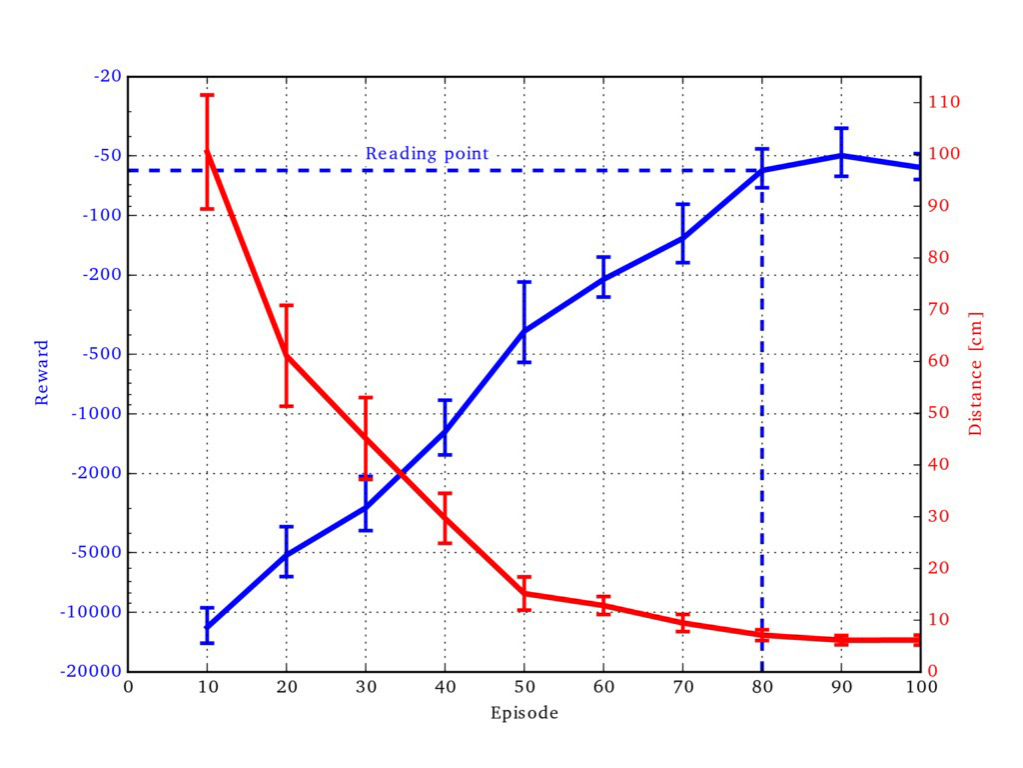
Learning curve of contextual policy search (BO-CPS and ES) in the ball-throwing domain. The left-sided logarithmic y scale represents the mean reward and the right-sided linear y scale represents the mean distance between the touchdown position and the given context. The learning curves indicate the mean and the corresponding SE over 16 test contexts. The reading point indicates where all final results are measured.

A learning curve is given in Figure 5. The average of the reward (negative squared distance to the target) and distance to the target over the test contexts are displayed. We noticed that after about 80 episodes the performance will not increase much more. We measured the final performance after 80 episodes in five experiments per acquisition function. The average measured distance to the test targets are 13.46, 15.24, 22.62, 17.40 and 29.99 cm (mean 19.74 cm, median 17.4 cm) for upper confidence bound and 6.54, 14.74, 6.68, 8.96 and 8.15 cm (mean 9.01 cm, median 8.15 cm) for entropy search. This evaluation clearly shows that it is possible to generalize throwing movements to a large area in only 80 episodes so that the average deviation from the target is almost at the maximum achievable precision. In addition, the computationally more complex entropy search shows consistently better results than upper confidence bound.

## 5. Application of the Learning Platform in Different Scenarios

Besides the evaluation of the learning platform in the ball-throwing scenario, it has been applied in different scenarios to transfer movements to different robotic systems. In a pick-and-place scenario a grasping movement was extracted from demonstrations of picking a box from a shelf, placing it on a table and putting it back afterwards. The movements were, similarly to the ball-throwing task, recorded with a marker based tracking system as detailed in (Gutzeit and Kirchner, 2016). After successful segmentation and classification of the grasping movement [see Gutzeit and Kirchner, 2016], it was imitated using a Cartesian space DMP and adapted to be executed on a Kuka iiwa lightweight robot equipped with a 3-finger gripper from Robotiq. Only one demonstration was required to learn the grasping movement. Cartesian DMPs were used for easy integration with the used whole-body control and perception. In this scenario the refinement was done using the CMA-ES algorithm in simulation. After 50–100 iterations, the movement could be successfully transferred to the robotic system. One demonstration of the initial trajectory is usually sufficient.

In another scenario, the learning platform was used to teach the robotic system Mantis (Bartsch et al., 2016) to pull a lever. Again, the recorded movements could be successfully segmented. Due to the fixed lever position in this experiment, the movement execution of the human was strongly predetermined leading to good classification results with only one training example per class (de Gea Fernndez et al., 2015; Gutzeit et al., 2018). Like in the pick-and-place scencario, RL techniques implemented in the learning platform were used to adapt the demonstrated movement to the robotic system. REPS and CMA-ES gave good results. After several hundred episodes in simulation, a successful movement could be generated. Learning could be done in parallel from multiple demonstrations with each RL learning process being initialized with a single demonstration.

As in the ball-throwing scenario, the transfer of the demonstrated movements was partially automated. To recognize the important movement segment, only a few manually labeled training examples are needed. To imitate and adapt the demonstrations to the system, the embodiment mapping and a reward function have to be selected with regards to the robotic system and the task goal. Besides this, the transfer of the movement to the system is completely automated.

Videos of these two applications of the learning platform can be found online.^3^

## 6. Conclusion and Outlook

Our results show that it is possible to learn new skills for robots without specifying the solution directly. The learning platform leverages intuitive knowledge from humans that do not know anything about the target system to automatically transfer skills to robots. The main impediments that can be overcome by the learning platform in this setting are the kinematic and dynamic differences of the demonstrator and the target system.

In this work, we usually learn reference trajectories that can be used for example in a whole-body control framework (de Gea Fernndez et al., 2015). The integration of more sensor data like camera images or force sensor measurements would be a next step.

The presented approach still has some limitations: some prior knowledge has to be defined in form of reward functions, simulations, and markers for motion capture. For complex problems with complex reward functions, learning the reward function would be better than defining it manually. Promising fields of research are active reward learning (Daniel et al., 2015) and inverse reinforcement learning (Ng and Russell, 2000). Simulations ideally would be created automatically from sensor data, experience, and active exploration in the real world. At the moment, however, this is still a manual step. For automated behavior recording, marker free approaches could be tested and compared with respect to accuracy and achievable automation level. Also some prior knowledge is implicitly integrated in the design of the learning platform. There is not one combination of methods that works for all applications. For example, simulation-reality-transfer is only required in challenging applications like ball-throwing. This should be addressed in future work.

On the basis of the learning platform, our future goal is to build a library of movements that are represented independent of the target system. We could use methods for embodiment mapping to transfer those skills to several target systems.

## Ethics Statement

Experimental protocols were approved by the ethics committee of the University of Bremen. Written informed consent was obtained from all participants that volunteered to perform the experiments. Written informed consent for publication of identifying information/images was also obtained from all participants.

## Author Contributions

LG and AF were responsible for the concept of the paper and are main authors. LG, AF, and MO wrote Section 2 and 3. LG organized the data acquisition, implemented and evaluated the behavior segmentation and annotation, and wrote Sections 2.1, 2.2 and 5. AF implemented imitation learning and reinforcement learning approaches, developed contextual policy search and active learning methods, and wrote Sections 2.3 and 2.4. MO implemented methods to transfer motion plans from simulation to reality, conducted experiments to evaluate these methods, and wrote Section 2.5. JHM implemented imitation learning methods, developed contextual policy search and active learning methods and wrote the introduction and Sections 2.4.2 and 2.4.3. JH conducted the experiments to evaluate BO-CPS on the robot COMPI and wrote Section 6. EAK and FK wrote the introduction, conclusion, and outlook.

## Conflict of Interest Statement

The authors declare that the research was conducted in the absence of any commercial or financial relationships that could be construed as a potential conflict of interest.

## Footnotes

1 For more details, see http://robotik.dfki-bremen.de/en/research/projects/besman.html

2 For more details, see http://www.qualisys.com/software/qualisys-track-manager/

3 Grasp box: https://robotik.dfki-bremen.de/de/mediathek/videoarchiv/besman-zweite-demo.html; pull lever: https://robotik.dfki-bremen.de/de/mediathek/videoarchiv/besman-dritte-demo.html
